# Hepatic fibrosis extracellular matrix stiffness induces abnormal maturation of dendritic cell via AMPK inhibition-mediated cholesterol accumulation

**DOI:** 10.1016/j.mtbio.2026.103606

**Published:** 2026-09-01

**Authors:** Xianlin Zeng, Pan Luo, Cuifang Wu, Jinhua Long, Shuai Zhang, Yun Wang, Lijing Teng, Honghong Zhang, Deqiao Fan, Zuquan Hu, Pu Xu, Zhu Zeng

**Affiliations:** a State Key Laboratory of Discovery and Utilization of Functional Components in Traditional Chinese Medicine, School of Basic Medical Sciences/School of Biology & Engineering, Guizhou Medical University, Guiyang, 561113, PR China; b Key Laboratory of Microbio and Infectious Disease Prevention & Control of Guizhou, Guiyang, 561113, PR China; c Key Laboratory of Infectious Immune and Antibody Engineering of Guizhou Province, Engineering Research Center of Cellular Immunotherapy of Guizhou Province, Guiyang, 561113, PR China; d Department of Oncology, Affiliated Hospital of Guizhou Medical University, Guiyang, 550004, PR China; e Department of Oncology, Affiliated Cancer Hospital of Guizhou Medical University, Guiyang, 550004, PR China

**Keywords:** Hepatic fibrosis, Extracellular matrix stiffness, Dendritic cells, Cholesterol metabolism, AMP-activated protein kinase

## Abstract

Dendritic cell (DC)-based immunotherapy shows limited efficacy against hepatic fibrosis, and the underlying mechanometabolic crosstalk remains unclear. Here, from a mechanobiological perspective, we demonstrate that increased extracellular matrix (ECM) stiffness in the fibrotic liver triggeres antigen-independent abnormal DC maturation, characterized by elevated co-stimulatory molecules, impaired phagocytic capacity, reduced IL-10 secretion, and suppressed regulatory T cell (T_reg_) differentiation. Mechanistically, the stiff ECM inhibites the AMPK-LXRα-ABCG1 signaling axis in DCs, reducing cholesterol efflux and promoting intracellular cholesterol accumulation. Cholesterol depletion or AMPK activation reverses stiffness-induced abnormal DC maturation. In mouse models, combined treatment with pirfenidone and simvastatin reduced liver stiffness and collagen deposition, and restored immune tolerance by correcting DC cholesterol metabolism. Our study identifies a mechanometabolic pathway linking matrix stiffness to DC dysfunction, providing a promising immunometabolic strategy for antifibrotic therapy.

## Graphical abstract

Mechanism of abnormal DC maturation induced by ECM stiffness via AMPK inhibition-regulated cholesterol accumulation. In hepatic fibrosis, increased ECM stiffness suppresses the AMPK–LXRα–ABCG1 signaling axis in DCs, thereby impairing cholesterol efflux and promoting intracellular cholesterol accumulation. This metabolic cascade induces abnormal DC maturation, inhibites T_reg_ differentiation, and disruptes hepatic immune homeostasis, ultimately exacerbating hepatic fibrosis.

**Figure:**
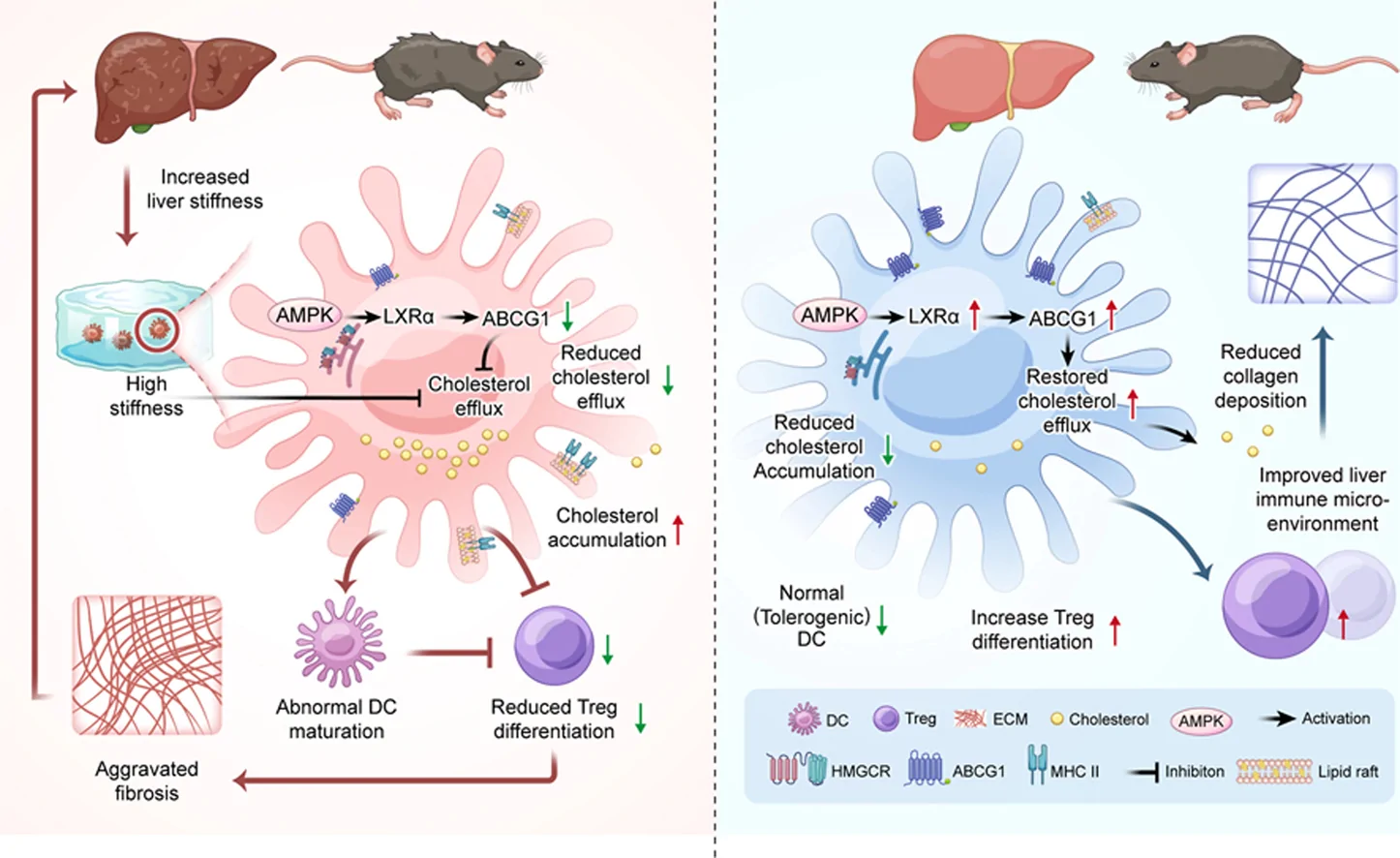

## 1 Introduction

Hepatic fibrosis, a wound-healing response to chronic liver injury, is characterized by excessive extracellular matrix (ECM) deposition and disruption of immune microenvironment homeostasis [1,2]. This pathological remodeling reshapes the hepatic mechanical microenvironment, creating a progressively stiffened tissue niche. Emerging evidence indicates that anti-HBV immunotherapy targeting DCs effectively attenuates hepatic fibrosis [3]. However, the heterogeneity of the mechanical microenvironment significantly increases the difficulty of treating non-viral hepatic fibrosis [4,5]. Currently, a key therapeutic bottleneck in hepatic fibrosis lies in the limited mechanistic understanding of how biomechanical cues regulate the functional heterogeneity of immune cells and their associated mechanotransduction mechanisms. Therefore, exploring cellular mechanobiological responses holds great promise for improving antifibrotic therapy.

The dysregulated hepatic immune microenvironment, particularly the imbalance of key immune cells including regulatory T (T_reg)_ cells, natural killer (NK) cells, and antigen-presenting cells (APCs), contributes critically to sustained inflammation and fibrogenesis [[6], [7], [8], [9]]. As the most potent APCs in the body, DCs play a pivotal role in orchestrating the balance between immune tolerance and inflammation in the liver. In the healthy livers, DCs maintain a low-activation state, which helps preserve hepatic immune tolerant microenvironment [[10], [11], [12]]. In the fibrotic liver , DCs exhibit an abnormally mature phenotype. These DCs can recognize damage-associated molecular patterns (DAMPs) released from injured hepatocytes and activate NF-κB signaling, thereby promoting hepatocyte apoptosis and inflammatory cascades [13]. Furthermore, DCs secrete interleukin-6 (IL-6) and tumor necrosis factor-α (TNF-α) to drive fibroblast proliferation and excessive ECM deposition [14,15]. Dysfunctional DCs can also promote aberrant T cell expansion, aggravating chronic inflammation and fibrogenesis [16,17]. Despite their central role, the upstream triggers and regulatory mechanisms underlying abnormal DC maturation during hepatic fibrosis remain poorly understood.

Increasing evidence indicates that the ECM is not a passive scaffold but an active regulator of immune function [18,19]. In particular, progressive ECM deposition and cross-linking during fibrosis lead to increased matrix stiffness, a pathological hallmark that closely correlates with the disease stage. DCs are highly plastic, as their immune-phenotype and function can dynamically change in response to mechanical stimuli from ECM [20,21]. For instance, DCs cultured on stiff substrates (50 kPa) exhibit enhanced expression of co-stimulatory molecules CD80, CD86 and altered cytokine secretion compared with those on compliant matrices (2 kPa) [22]. However, the molecular mechanisms by which fibrosis-associated ECM stiffness modulates DC function in the liver remain largely undefined.

Cholesterol, an essential lipid component of cellular membranes, plays a fundamental role in immune signaling by regulating the organization of lipid rafts and the assembly of immune receptors [23,24]. Cholesterol-rich membrane domains facilitate the clustering of peptide–MHC II complexes on APCs and enhance T cell activation [25,26]. Crucially, cholesterol metabolism is closely associated with the functional remodeling of DCs. For instance, hypercholesterolemia-induced cholesterol accumulation in DCs augments their antigen-presenting capability and T cell proliferative potential, increasing the risk of autoimmune diseases [27,28]. These findings establish cholesterol metabolism as a critical determinant of DC activation state. However, it remains unclear whether fibrosis-associated ECM stiffness drives persistent DC-mediated inflammatory responses by modulating intracellular cholesterol homeostasis.

In this study, we established a 3D hydrogel system simulating the pathological stiffness of hepatic fibrosis and demonstrated that hepatic fibrosis was associated with a shift toward abnormal DC maturation. Fibrotic matrix stiffness drove dysfunction of DCs by inducing intracellular cholesterol accumulation. Mechanistically, we revealed that AMPK inhibition impairs DC function by modulating cholesterol metabolism. Critically, pharmacological inhibition of cholesterol synthesis restores the immunoregulatory function of DCs. Furthermore, the animal experiments confirmed that inhibiting collagen deposition and cholesterol accumulation alleviated hepatic fibrosis. Collectively, our findings suggest that targeting DC cholesterol metabolism is a promising strategy to enhance the clinical effectiveness of anti-fibrotic therapy.

## 2 Materials and methods

### 2.1 Single-cell sequencing analysis

The original datasets were sourced from GSE136103. This cohort contained a total of five human liver tissue samples obtained from patients with liver cirrhosis and non-fibrotic healthy controls. All raw sequencing data underwent standardized preprocessing and quality control filtering steps: cells with fewer than 200 detected genes, cells harbouring >20% mitochondrial reads, and potential multiplet cells were discarded to eliminate low-quality events. All downstream analyses were performed using the Seurat (v4.3.01) software package in R. After quality control, the gene expression was normalized via the Seurat "LogNormalize" method. The integrated expression matrix was scaled, and principal component analysis (PCA) was conducted for dimensional reduction. Distinct cell subpopulations were annotated using the SingleR package in R. Gene sets associated with cholesterol metabolism were used to score DC subtypes via the AUCell algorithm.

### 2.2 Animals and reagents

Male C57BL/6J mice (6 weeks old) were purchased from the Experimental Animal Center of Guizhou Medical University. All animal experiments were conducted in accordance with the protocols approved by the Ethics Committee of Guizhou Medical University (No. 2100066). Olive oil, carbon tetrachloride (CCl_4_), methyl-β-cyclodextrin (M-βCD), BODIPY-Cholesterol, simvastatin, and pirfenidone (PFD) were purchased from MedChemExpress (MCE, Shanghai, China). Flow cytometry antibodies CD11c, CD3, CD4, CD8, CD25, CD40, CD45, CD80, CD86, MHC II, IL-10, IL-12, and Foxp3, along with protein transport inhibitors, fixation/permeabilization solutions, TRIzol reagent, and lipid raft labeling kits, were obtained from Thermo Fisher Scientific Inc. (Shanghai, China). Murine GM-CSF and murine IL-4 were purchased from PeproTech (Suzhou, China). CD4^+^ T cell isolation kits, LS columns, and MACS Rinsing Solution were obtained from Miltenyi Biotec (Shanghai, China). ELISA kits for IL-6, IL-10, TNF-α, IFN-γ, IL-1β, and MMP-9 were purchased from MultiSciences Biotech (Hangzhou, China). FITC-labeled dextran was obtained from Sigma-Aldrich (Shanghai, China). Antibodies against HMGCR, ABCG1, GAPDH, and goat anti-rabbit IgG secondary antibodies were purchased from HuaAn Biotechnology (Hangzhou, China). The Amplex Red Cholesterol Assay Kit was obtained from Beyotime Biotechnology (Shanghai, China).

### 2.3 Establishment and treatment of mouse models of liver fibrosis

A mouse model of liver fibrosis was constructed by intraperitoneal injection of 20% carbon tetrachloride (CCL_4_) diluted in corn oil at a dose of 5 μL/g body weight, administered twice weekly for 12 weeks. Age-matched control mice received an identical regimen of olive oil injection, serving as vehicle controls to rule out solvent-specific hepatic effects. Fibrotic mice were randomly divided into four groups and treated daily with 200 μL PBS (by gavage), 200 mg/kg PFD (by gavage), and/or 10 mg/kg simvastatin (by intraperitoneal injection twice a week). Samples were collected after 4 weeks of treatment.

### 2.4 Isolation and flow cytometric analysis of liver immune cells

Fresh liver tissues were minced and passed through a 70 μm cell strainer to obtain single-cell suspensions. Liver immune cells were isolated using 40% Percoll density gradient centrifugation. After treatment with a protein transport inhibitor for 4 h, cells were fixed and permeabilized, followed by incubation with specific antibodies (CD11c, MHC II, CD40, CD80, CD4, CD25, Foxp3) for 20 min at room temperature. To ensure the accuracy of flow cytometric analysis, strict experimental controls and standardized gating strategies were applied and maintained consistently across all treatment groups. Single-stained compensation beads (BD Pharmingen, 552845) were utilized to establish the fluorescence compensation matrix and correct for spectral overlap. Corresponding isotype controls were employed to accurately determine background fluorescence and establish precise gating boundaries for positive populations. The detailed gating strategies for *in vivo* and *in vitro* experiments are illustrated in Supplementary Fig. S2A and S2D, respectively. following the manufacturer's protocol prior to sample acquisition. Data were acquired using a flow cytometer (BD Biosciences, FACSCanto II) and analyzed with FlowJo software.

### 2.5 Liver function testing

Serum levels of aspartate aminotransferase (AST) and alanine aminotransferase (ALT) were measured using commercial assay kits (Solarbio, Beijing, China) according to the manufacturer's instructions.

### 2.6 Histological analysis and immunofluorescence staining

Fresh liver tissues were fixed in 4% paraformaldehyde for 24 h, followed by dehydration, permeabilization, embedding, and sectioning to obtain paraffin sections. HE, Masson, and Sirius Red staining were performed according to manufacturer guidelines. Visualization was performed using a panoramic tissue micro-imaging system to evaluate immune cell infiltration and collagen deposition.

For immunofluorescence, paraffin sections were deparaffinized, rehydrated, and subjected to antigen retrieval. Samples were blocked with bovine serum albumin for 30 min and incubated with specific antibodies (CD11c, MHC II, HMGCR) at 4°C overnight, followed by incubation with fluorescent secondary antibodies for 2 h at room temperature. Fluorescent images were acquired on a confocal laser scanning microscope.

For quantitative analysis, three randomly selected, non-overlapping fields of view were imaged for each tissue section. The mean fluorescence intensity (MFI) of HMGCR was measured using ImageJ to determine relative HMGCR expression levels. Colocalization between CD11c and HMGCR signals was quantified with ImageJ software. Briefly, linear regions of interest (ROIs, denoted by red/yellow lines in corresponding figures) were manually drawn across DC-rich areas along each section; Manders’ overlap coefficients were calculated within these defined ROIs to evaluate the degree of protein colocalization.

### 2.7 Stiffness assays

Liver tissue stiffness was measured by atomic force microscopy (AFM) and rheometry. Before AFM measurements, frozen tissue blocks were cut into 50 μm thick sections. Each section was submerged in PBS at room temperature. We used silicon nitride cantilevers (Bruker, USA, MLCT O10) with a spring constant of 0.07 N/m. The cantilever was calibrated using the thermal noise method before each experiment. The Young's modulus of tissue was determined using the Hertz model. Before rheological measurements, fresh liver tissue samples were obtained using a mouse skin punch with a diameter of 8 mm, and each sample was immersed in PBS. Measurements were performed using a parallel plate geometry (8 mm diameter) with shear strain of 0.5% and a frequency of 1 Hz. The compressive modulus and storage modulus were recorded over time.

Material stiffness was measured using a rheometer (MCR 302e, Anton Paar) with a 20 mm diameter parallel plate. The relationship between UV gelation and irradiation time was determined by shear rheology. Measurements were conducted at shear strain of 0.5%, frequency of 1 Hz, and UV power of 25 W. The compressive modulus and storage modulus were monitored for 120 s during UV exposure, and a UV time-scan curve was generated.

### 2.8 Hydrogel preparation

Hydrogels were synthesized following published protocols with minor adjustments. Briefly, 120 mg GelMA plus 16 mg OHA were dissolved in 4 mL complete medium to generate low-stiffness (LS, 100 Pa) hydrogels, while 200 mg GelMA and 24 mg OHA in the same medium yielded high-stiffness (HS, 1000 Pa) formulations. All constructs were UV-crosslinked, and their mechanical stiffness was quantified as storage modulus (G′) via rheometry.

### 2.9 In *vitro* generation and functional assays of bone marrow-derived DCs

Bone marrow-derived precursor cells were isolated and cultured with GM-CSF and IL-4 for 6 days to generate immature DCs (imDCs). Following differentiation, the harvested imDCs were seeded into prefabricated LS or HS hydrogel and incubated for an additional 48 h to receive matrix-derived mechanical cues. To assess maturation status, cells were fixed in 4% paraformaldehyde for 15 min at room temperature. After washing, cells were stained with antibodies targeting MHC II, CD40, CD80 and CD86 for 20 min at room temperature. Fluorescence compensation was pre-calibrated using BD compensation beads as detailed above, and marker expression was quantified by flow cytometry. To evaluate antigen phagocytic capacity, FITC-dextran was added and incubated at 37°C for 4 h. Parallel cells were incubated with identical dextran solutions at 4°C to suppress energy-dependent endocytosis and record non-specific surface binding background. The mean fluorescence intensity (MFI) of FITC in DCs was measured by flow cytometry. During flow cytometric analysis, the positive gating threshold was strictly defined based on a 4°C cold inhibition control group. The gate was set at the uppermost 0-1% of fluorescence events of the 4°C population to rigorously exclude false-positive signals from non-specific membrane binding, ensuring that only cells with genuine active intracellular FITC-dextran internalization were quantified. To evaluate DC-mediated CD4^+^ T cell priming, naïve CD4^+^ T cells were sorted from mouse spleens using magnetic beads and co-cultured with treated DCs for 48 h. Data were collected by flow cytometry and analyzed with FlowJo.

### 2.10 ELISA

DCs were seeded in hydrogels of different stiffness and treated for 48 h. Culture supernatants were collected, and levels of IL-6, IL-10, TNF-α, IFN-γ, IL-1β, and MMP-9 were measured using ELISA kits according to the manufacturer's instructions.

### 2.11 Mechanical characterization of DCs

DCs cultured in hydrogels of different stiffness were exposed to culture media with varied osmotic pressure (295 mOsm/kg, 235 mOsm/kg, 175 mOsm/kg, 115 mOsm/kg and 85 mOsm/kg) for 1 h. The cell viability was detected to evaluate cellular mechanical tension, which reflects the capacity of DCs to resist hypotonic pressure.

Membrane fluidity was determined by fluorescence polarization using 1,6-diphenyl-1,3,5-hexatriene (DPH) staining [11]. Fluorescence polarization *P* values were recorded, where lower *P* values indicate increased membrane fluidity.

### 2.12 RNA-Seq analysis

Total RNA was extracted from DCs treated with different stiffness hydrogels using TRIzol reagent. Sequencing libraries were generated by Majorbio Bio-Pharm Technology using an Illumina® Stranded mRNA Prep kit and sequenced on a NovaSeq X Plus platform.

### 2.13 Measurement of cholesterol levels

DCs were seeded in hydrogels of different stiffness and treated for 48 h. After washing three times with PBS, cells were labeled with 1 mL BODIPY-Cholesterol for 20 min at 37°C, washed again, and fixed with 4% paraformaldehyde for 10 min. Cells were then stained with rhodamine-labeled phalloidin and mounted with anti-fade mounting medium containing DAPI. Cholesterol expression and distribution were observed using a confocal laser scanning microscope.

To quantify cholesterol content, 1 × 10^7^ treated DCs were collected, lysed with isopropanol, and centrifuged at 500 × g for 5 min. Supernatants were collected, and intracellular total cholesterol and free cholesterol levels were detected using assay kits (Beyotime) according to the manufacturer's instructions.

### 2.14 Quantitative real-time PCR (qPCR)

Total cellular RNA was isolated from DCs using TRIzol reagent and reverse-transcribed into cDNA using a reverse transcription kit. RT-qPCR was performed using SYBR Green Master Mix on a Bio-Rad CFX96 Real-Time PCR System, with GAPDH used as an internal reference for normalization. The primers used in this study are listed in Table S1.

### 2.15 Western blot analysis

Treated DCs were lysed using RIPA buffer (Solarbio), and supernatants were collected by centrifugation to obtain protein samples. Proteins were separated by SDS-PAGE and transferred onto PVDF membranes (Millipore). Membranes were blocked with rapid blocking buffer for 10 min and incubated with primary antibodies (anti-GAPDH, anti-HMGCR, anti-ABCG1, anti-AMPK1, anti-AMPK2, anti-LXR) at 4°C overnight, followed by incubation with secondary antibodies for 1 h at room temperature. Protein bands were visualized using an Ultra High Sensitivity ECL Kit (MCE), and densitometric analysis was performed using ImageJ software.

### 2.16 Statistical analysis

Data analysis was performed using GraphPad Prism software (version 10). Comparisons between two groups were analyzed using an unpaired two-tailed Student's t-test, and comparisons among multiple groups were analyzed using one-way ANOVA. Pearson correlation analysis was performed to evaluate linear associations between liver stiffness and intrahepatic immune parameters. Differences were considered statistically significant at *P* < 0.05.

## 3 Results and discussion

### 3.1 DCs tended to maturation in fibrotic livers

Increased tissue stiffness is a hallmark and potential therapeutic target of hepatic fibrosis [29,30]. As a critical biomechanical signal, stiffness modulates cellular functions and reshapes the hepatic immune microenvironment. DCs serve as central regulators of hepatic immune homeostasis [17,31]. However, the mechanisms by which fibrotic stiffness regulates DC differentiation and function remain unclear.

**Fig. 1:**
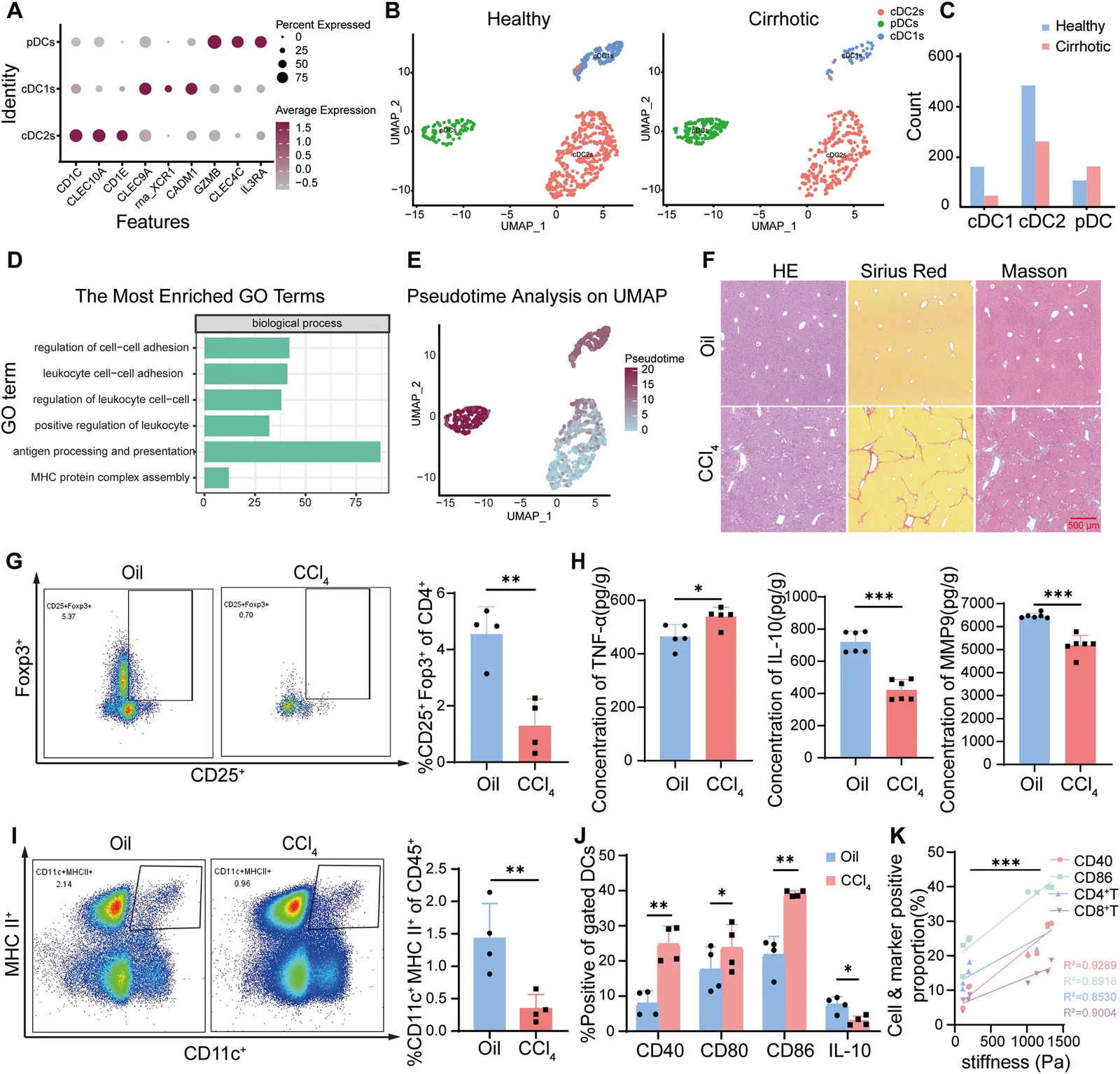
Phenotypic and Functional Characteristics of DCs in Hepatic Fibrosis. (A) Annotation of DCs subsets in liver tissues by single-cell RNA sequencing. Dot size represents the fraction of cells expression the genes, and color indicates mean expression levels. (B) t-SNE/UMAP plot showing the distribution of DCs in liver tissues. (C) Quantification of DCs subsets in liver tissues. (D) GO enrichment analysis of DEGs in DCs from liver tissues. (E) Pseudotime trajectory analysis of DCs. (F) Representative images of HE, Masson and Sirius Red staining of liver. (G) Proportion of T_reg_ cells in liver tissues. (H) Concentrations of TNF-α, IL-10, and MMP-9 in liver tissues. (I) Proportion of DCs in liver tissues. (J) Expression levels of CD40, CD80, CD86, and IL-10 in hepatic DCs (All values represent the percentage of marker-positive cells pre-gated on CD45^+^CD11c^+^MHC II^+^ hepatic DCs). (K) Correlation analysis between liver stiffness and immune molecules in mice (Data include percentages of DC maturation markers (CD40, CD86) and frequencies of CD4^+^/CD8^+^ T cells. Each data point represents an independent biological replicate). For the graph: Mean ± SD, n = 4 to 6 in each group. *P < 0.05; **P < 0.01; ***P < 0.001. (For interpretation of the references to color in this figure legend, the reader is referred to the Web version of this article.)

To elucidate the phenotypic and functional characteristics of DCs in hepatic fibrosis, we first identified three subtypes of DCs from hepatic immune cells of patients with advanced liver fibrosis (cirrhosis) based on specific marker genes (Fig. S1 and Fig. 1A). The proportion of conventional DCs (cDCs) in the fibrotic livers was significantly lower than that in normal livers (Fig. 1B and C). Furthermore, KEGG enrichment analysis revealed that differentially expressed genes (DEGs) in these DCs were enriched in antigen-presenting pathways (Fig. 1D). Pseudo-time trajectory analysis confirmed that cDC1 exhibited higher maturity than cDC2 (Fig. 1E). Given that DC maturation during hepatitis progression can induce CD8^+^ T cell exhaustion and disrupt T_reg_-mediated immune tolerance [32,33], these results suggest that abnormal DC number and function exacerbate liver immune disorders driven by chronic inflammation and thereby promote fibrogenesis.

To validate these single-cell RNA sequencing results, we established a CCl_4_-induced mouse model of liver fibrosis. Fibrotic mice exhibited rough granular liver surfaces, extensive collagen deposition (Fig. 1F), and elevated serum Aspartate Aminotransferase (AST) and Alanine Aminotransferase (ALT) levels (Fig. S1). These results confirmed the successful establishment of the liver fibrosis model. Immunophenotyping of immune cells in liver tissues revealed a significant accumulation of CD4^+^ and CD8^+^ T cells alongside reduced proportions of CD25^+^Foxp3^+^ T_reg_ (Fig. S2 B and Fig. 1G). This imbalance between pro-inflammatory effector T cells and immunosuppressive T_reg_ aligns with established immune mechanisms underlying chronic liver fibrosis. Previous work has demonstrated that persistent hepatocellular injury drives clonal expansion of cytotoxic CD8^+^ and helper CD4^+^T cells [34,35]. Meanwhile, the inflammatory niche restricts T_reg_ recruitment and impairs anti-inflammatory IL-10 secretion, ablating their capacity to constrain pro-fibrotic inflammation [[36], [37], [38]]. Consistent with this immune dysregulation, our results show elevated levels of TNF-α and IL-6, alongside reduced anti-inflammatory IL-10 in fibrotic liver homogenates (Fig. 1H).

Furthermore, we observed that the proportion of DCs (CD45^+^CD11c^+^MHCII^+^) decreased in fibrotic liver tissues, surface molecules CD40, CD80, and CD86 were significantly increased, and the frequency of IL-10-producing DCs was reduced (Fig. 1J). These observations align with the immunoregulatory role reported by Andrews et al. [39,40] and further support the model proposed by HU et al., in which mature DCs activate hepatic stellate cells and inhibit T_reg_ differentiation via TNF-α secretion, leading to insufficient production of anti-inflammatory factors such as IL-10 and ultimately promoting hepatic fibrosis [12]. Notably, the 0-2.5% frequency of hepatic DCs detected in our study is consistent with standardized immune profiling workflows and published fibrosis datasets [41,42], confirminghepatic DCs as a rare yet functionally critical myeloid subset within liver immunity. Our human single-cell data were derived from cirrhotic patients, and the *in vivo* mechanistic analyses were conducted at a single 12-week endpoint that recapitulates advanced fibrotic pathology [43,44]. Consequently, the stiffness-dependent DC maturation described herein primarily reflects phenotypic changes occurring in late-stage liver fibrosis. Future longitudinal studies incorporating multiple time points are warranted to determine whether these DC phenotypic adaptations develop progressively throughout fibrogenesis or emerge specifically at advanced disease stages.

A central challenge in mechanobiology is to recapitulate in *vivo* mechanical environment in *vitro*. Hepatic stiffness varies considerably across measurement scales. Clinical transient elastography defines macroscopic thresholds: normal (<6 kPa), fibrotic (6–12.5 kPa), and cirrhotic (>12.5 kPa) [19,45]. In contrast, micromechanical measurements report much lower moduli, with healthy mouse liver at approximately 150 Pa [46,47]. To ensure robustness, this study employed two microscopic methods for cross-validation, thereby confirming the reliability of the fibrosis model. We found that the stiffness of normal mouse liver was 174.5 ± 56.8 Pa, whereas fibrotic liver stiffness increased to 1197.3 ± 554.2 Pa (Fig. S1). Crucially, correlation analysis demonstrated a robust positive association between hepatic stiffness and immune signals, including DC maturation markers (CD40, CD86), as well as CD4^+^ T and CD8^+^ T cell proportions (Fig. S2 B and Fig. 1K). These findings indicated that DCs tended toward maturation in fibrotic livers, and that immune activation and ECM stiffening changed synergistically during disease progression, providing mechanistic evidence linking liver stiffness to DC immune dysfunction.

### 3.2 Hepatic fibrosis ECM stiffness induced abnormal maturation of DCs

**Fig. 2:**
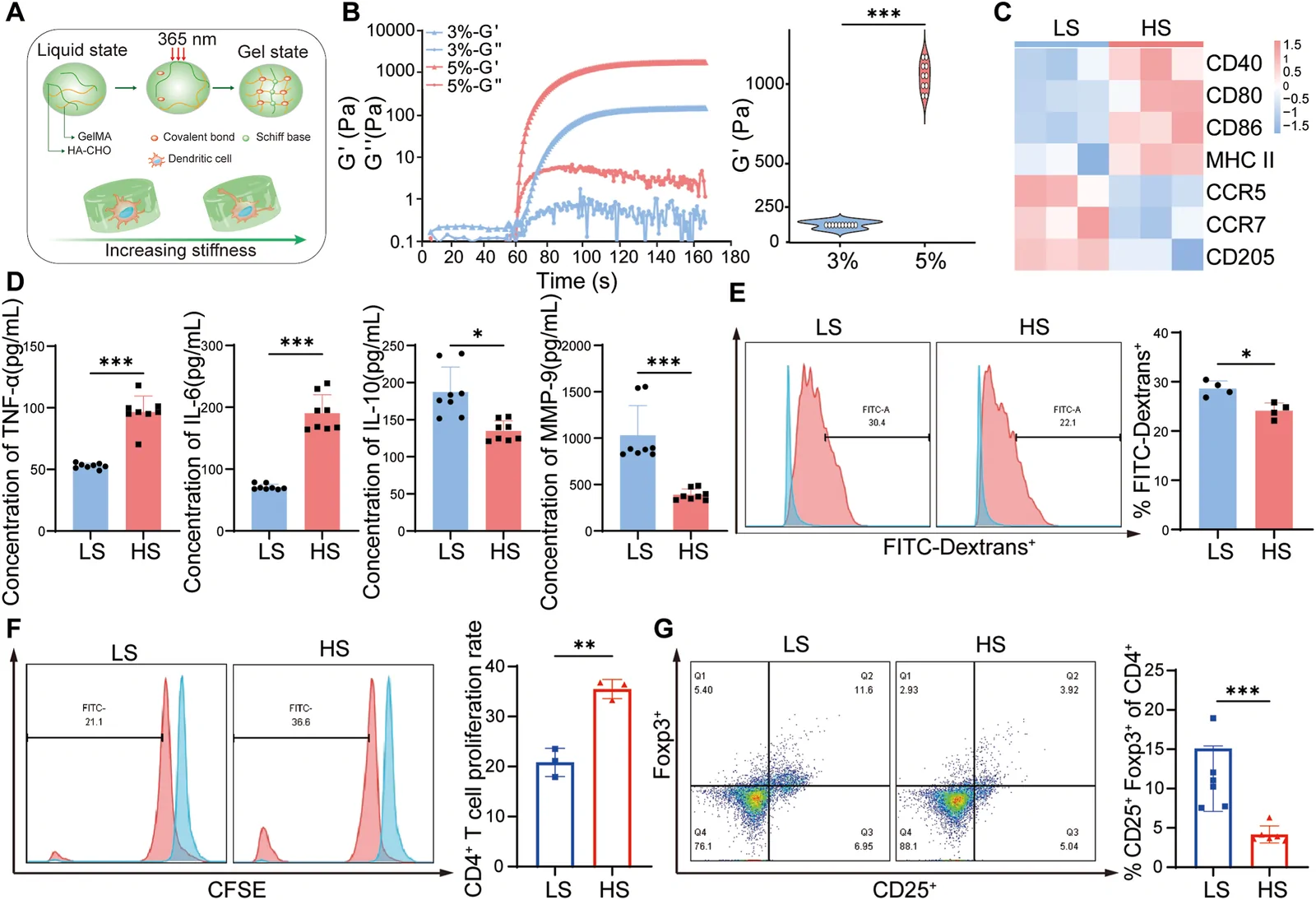
Hepatic Fibrosis Matrix Stiffness Induced Abnormal Maturation of DCs (A) Schematic illustration of the tunable hydrogel system mimicking ECM stiffness in hepatic fibrosis. (B) Quantification of hydrogel stiffness measured by rheometer (G': storage moduli, G″ compressive moduli). (C) Expression levels of surface molecules on DCs treated with different stiffness. (D) Cytokine secretion profiles of supernatants from DCs cultured under different stiffness. (E) Phagocytic capacity of DCs treated with different stiffness (Blue curve: 4°C unstained negative control; pink curve: DCs incubated with FITC-dextran for active phagocytosis detection. All fluorescence signals were normalized to the 4°C background signal for each independent replicate). (F) Effect of DCs treated with different stiffness on CD4^+^ T cell proliferation (the blue curve represents non-proliferating T cells, and the orange curve indicates T cells after 48 h of co-culture with DCs). (G) Effect of DCs treated with different stiffness on T_reg_ differentiation. For the graph: Mean ± SD, n = 3 to 8 in each group. **P < 0.01; ***P < 0.001. (For interpretation of the references to color in this figure legend, the reader is referred to the Web version of this article.)

To determine whether ECM stiffening drives the abnormal maturation of DCs during liver fibrosis, we prepared hydrogels with different stiffness using GelMA and OHA in specific ratios (Fig. 2A). Rheometer analysis showed that the stiffness values were approximately 100 Pa and 1000 Pa, respectively matching the measured stiffness of healthy and fibrotic liver tissues (Fig. 2B). Notably, native livers exhibit profound mechanical heterogeneity across lobes and microanatomical niches. Atomic force microscopy and magnetic resonance elastography have identified discrete stiff fibrotic scars in fibrotic livers [48,49]. Our uniform single-stiffness hydrogels cannot replicate this complex gradient architecture. This regional variability may lead to inconsistent DC mechanosensory responses *in vivo*, thereby limiting the generalizability of our single-stiffness *in vitro* findings to the entire hepatic DC population. Future studies incorporating tissue-engineered liver constructs with zoned stiffness or precision-cut liver slice cultures will be crucial to validate this mechano-metabolic axis within more physiologically relevant, heterogeneous mechanical microenvironments.

After 48 h of culture in high-stiffness hydrogels, DCs displayed a markedly mature phenotype marked by elevated expression of co-stimulatory molecules CD40, CD80, CD86, and MHC II, concomitant with downregulation of CCR5, CCR7, and the endocytic receptor CD205 (Fig. 2C). Additionally, DCs treated with high stiffness secreted higher levels of TNF-α and IL-6, but lower levels of IL-10 and matrix metalloproteinase-9 (MMP-9) (Fig. 2D). Further analysis of phagocytic capability revealed that high-stiffness-treated DCs exhibited impaired dextran internalization compared to low-stiffness-treated DCs (Fig. 2E, Fig. S3E). This phagocytic deficit aligns with the observed downregulation of CD205, a key endocytic receptor whose reduction directly compromises antigen-capturing capacity of DCs [50]. Conversely, DCs cultured under high stiffness promoted CD4^+^ T cell proliferation and stimulated co-cultured T cells to secrete pro-inflammatory cytokines, including IFN-γ, IL-1β, and TNF-α (Fig. 2F, Fig. S3). This effect may be attributed to the upregulated expression of MHC II, a central molecule for exogenous antigen presentation, which strengthens the interaction between DCs and CD4^+^ T cells and thereby facilitates T cell expansion [51].

In addition to driving effector T cell activation, high ECM stiffness significantly impaired the ability of DCs to induce T_reg_ differentiation (Fig. 2G). Although we have not delineated the signaling cascade connecting ECM stiffness to DC-dependent T_reg_ differentiation. Prior studies identify DC-secreted IL-10 as a key cytokine that sustains peripheral T_reg_ development and homeostasis [52,53]. Our data demonstrate that high ECM stiffness robustly suppresses DC IL-10 secretion, and this reduction in IL-10 may contribute to impaired T_reg_ differentiation within the fibrotic liver microenvironment. Furthermore, it is highly likely that other concurrent alterations, such as modified co-stimulatory signaling and the relative contribution of pro-inflammatory cytokines (e.g., TNF-α and IL-6), also play integral roles in restricting T_reg_ formation in this context. Future dedicated investigations using specific cytokine blockade will be necessary to precisely delineate the respective contributions of IL-10, pro-inflammatory cytokines, and dysregulated co-stimulatory molecules to this mechanosensitive DC-T_reg_ crosstalk. Collectively, the elevated ECM stiffness mimicking that of fibrotic liver tissue promotes abnormal DC maturation, characterized by upregulated co-stimulatory molecules, impaired phagocytosis, and reduced T_reg_ induction (Fig. 2). These observations are consistent with previous reports that stiffness promotes DC maturation and TNF-α secretion [54,55].

Additionally, Saleem et al. reported that increased cellular tension can reduce endocytic efficiency [56]. DC maturation is accompanied by membrane phospholipid remodeling and increased fluidity [57,58]. Consistent with these reports, our data showed that elevated ECM stiffness increases cellular tension and membrane fluidity in DCs (Fig. S3C and D). These biomechanical alterations provide a structural basis for the reduced phagocytosis and enhanced co-stimulatory marker expression observed in our model. Collectively, our results further establish ECM stiffness as a critical biomechanical signal governing aberrant DC immune function.

### 3.3 Hepatic fibrosis ECM Stiffness Promoted Cholesterol Accumulation in DCs

**Fig. 3:**
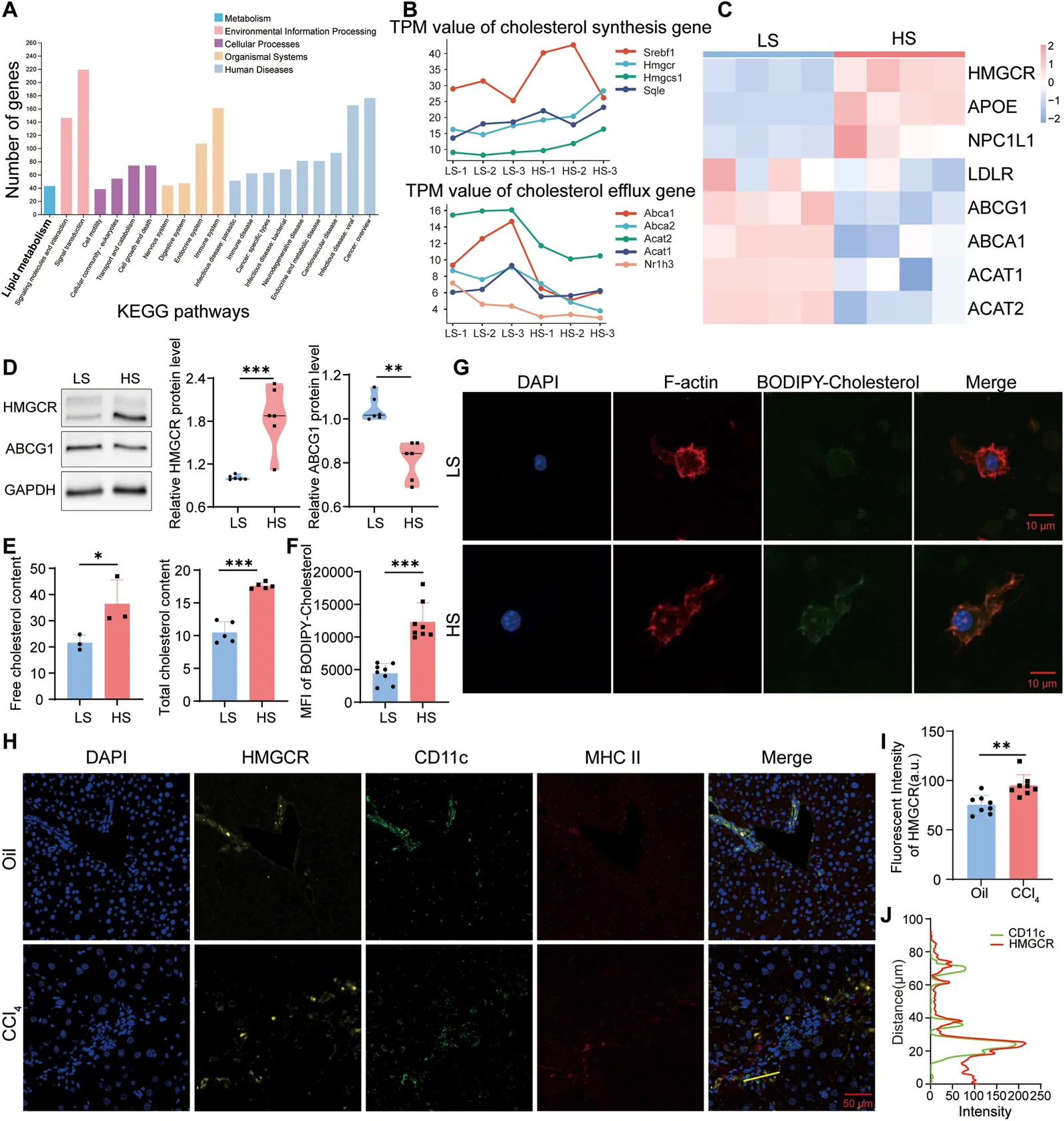
Liver Fibrosis Matrix Stiffness Promoted Cholesterol Accumulation in DCs. (A) KEGG enrichment analysis of DEGs in DCs treated with different stiffness. (B) Expression levels of cholesterol metabolism-related genes in DCs treated with different stiffness (LS-1 to 3 and HS-1 to 3 represent the corresponding three biological replicates of DCs cultured in different stiffness hydrogels). (C) mRNA expression levels of cholesterol metabolism-related genes in DCs. (D) Protein expression levels of cholesterol metabolism-related proteins in DCs. (E) Quantification of total cholesterol and free cholesterol levels in DCs treated with different stiffness (μmoL/10^8^ cells). (F, G) Representative immunofluorescence images showing cholesterol distribution and content in DCs treated with different stiffness (Blue: Nuclei; Red: F-actin; Green: Cholesterol probe BODIPY-Cholesterol). (H) Representative immunofluorescence images of mouse liver tissues (Blue: Nucleus; Yellow: HMGCR; Green: CD11c; Red: MHC Ⅱ). (I) Quantification of HMGCR fluorescence intensity in hepatic DCs (Each data point represents one biological replicate, and three randomly selected non-overlapping fields of view were quantified per biological sample). (J) Colocalization analysis of CD11c and HMGCR proteins (Yellow lines indicate the selected regions for quantitative colocalization calculation). For the graph: Mean ± SD, n = 3 to 8 in each group. *P < 0.05; **P < 0.01; ***P < 0.001. (For interpretation of the references to color in this figure legend, the reader is referred to the Web version of this article.)

To explore the mechanism of abnormal DC maturation caused by liver fibrosis ECM stiffness, we performed RNA sequencing (RNA-seq) on DCs cultured in 3D hydrogels with different stiffnesses. Transcriptomic profiling revealed that DEGs were enriched in pathways associated with lipid metabolism (Fig. 3A). Specifically, genes involved in cholesterol biosynthesis were upregulated, whereas those regulating cholesterol efflux were downregulated (Fig. 3B). Quantitative real-time polymerase chain reaction and Western blot consistently confirmed that high-stiffness treatment significantly increased DC expression of 3-hydroxy-3-methylglutaryl coenzyme A reductase (HMGCR), the rate-limiting enzyme in cholesterol synthesis, while suppressing ATP-binding cassette subfamily G member 1 (ABCG1), a key mediator of cholesterol efflux (Fig. 3C and D). Consistent with these results, the levels of total cholesterol and free cholesterol in high-stiffness-treated DCs were significantly higher than those in low-stiffness-treated DCs (Fig. 3E). Previous studies have reported that free cholesterol accumulation upregulates the expression of co-stimulatory molecules such as CD40 and CD80 on DCs [59], and our results are highly consistent with this notion, suggesting that cholesterol accumulation may serve as a critical metabolic basis for stiffness-driven abnormal DC activation.

As a key lipid molecule governing membrane fluidity, lipid raft assembly, and immune signal transduction in DCs [27,60], cholesterol distribution and accumulation directly dictate cellular function. The BODIPY-cholesterol staining revealed pronounced cholesterol accumulation specifically on the dendritic protrusions of high-stiffness-treated DCs (Fig. 3F and G). We found that fibrotic stiffness also elevated lipid raft content in DCs (Fig. S4 A). These observations align well with established mechanisms by which cholesterol modulates DC function, suggesting that cholesterol accumulation may contribute to stiffness-induced abnormal DC activation by regulating lipid raft assembly.

Importantly, similar metabolic alterations were observed in hepatic DCs from mice with hepatic fibrosis. Total cholesterol content was markedly higher in DCs from fibrotic mice than in those from healthy controls (Fig. 3H), accompanied by elevated HMGCR protein expression in hepatic DCs (Fig. 3I). These changes were fully consistent with the stiffness-induced metabolic reprogramming observed *in vitro*, confirming the *in vivo* relevance of stiffness-driven cholesterol accumulation in DCs. Notably, the stiffness-dependent DC phenotypic maturation, along with the observed cholesterol accumulation and metabolic alterations, primarily reflect the microenvironment of advanced fibrosis and cirrhosis. Therefore, these stiffness-triggered cellular and metabolic alterations cannot yet be extrapolated to the early phases of liver fibrosis.

Collectively, these findings suggest that increased ECM stiffness promoted cholesterol synthesis while inhibiting efflux, synergistically mediating cholesterol accumulation in DCs during hepatic fibrosis. This mechanism mirrors stiffness-mediated cholesterol accumulation reported in diverse tumor microenvironments [[61], [62], [63]], providing critical experimental support for delineating the metabolic regulatory network underlying aberrant DC immune function during hepatic fibrosis.

### 3.4 Cholesterol Depletion Effectively Reversed Stiffness-Induced Abnormal Maturation of DCs

**Fig. 4:**
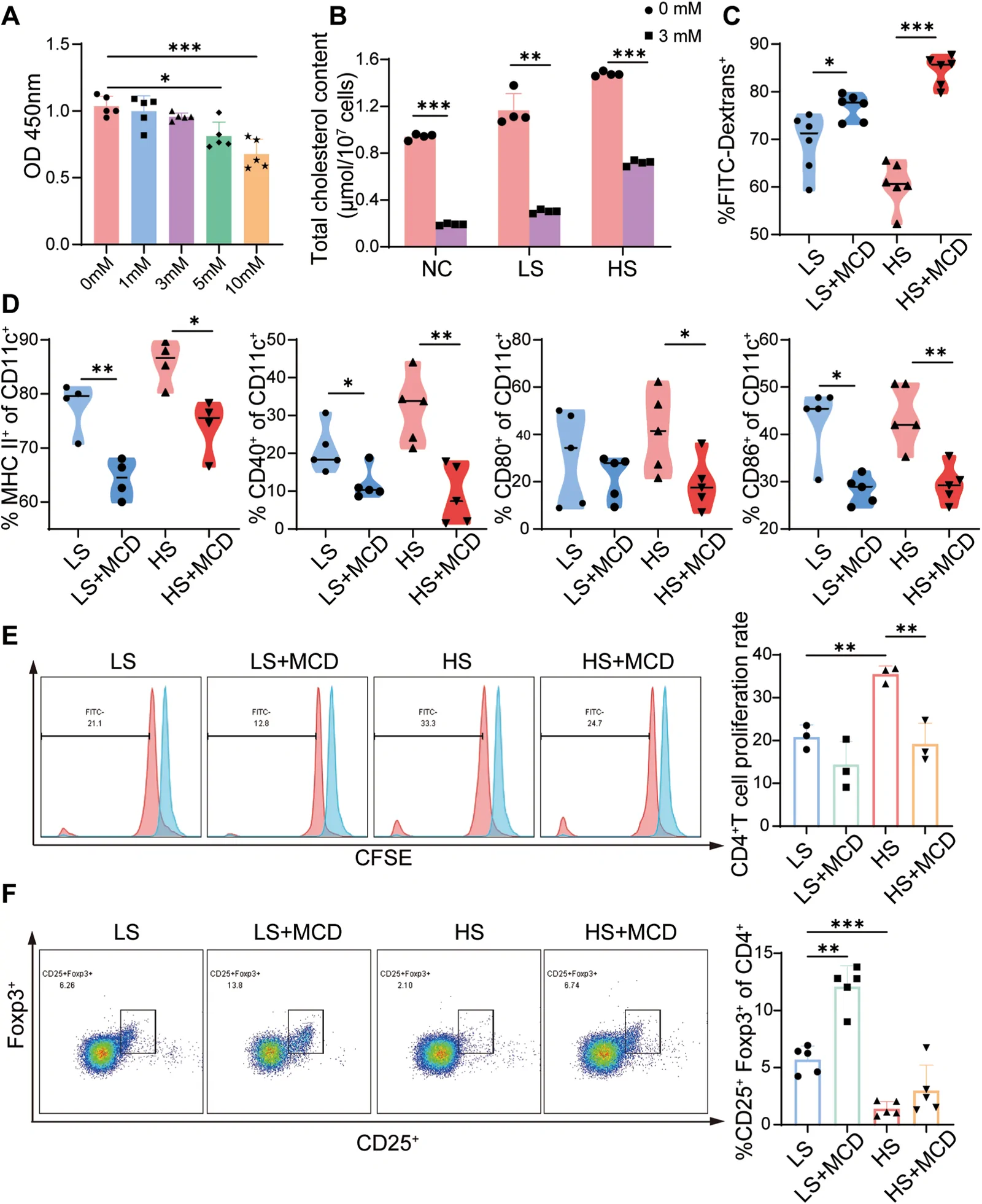
Cholesterol Depletion Effectively Reversed Stiffness-Induced Abnormal Maturation of DCs. (A) Effect of M-βCD treatment at different concentrations on DC viability (Cell viability was assessed via CCK-8 assay, with drug concentrations plotted on the x-axis). (B) Effect of M-βCD treatment on cholesterol content in DCs (sus is defined as suspension-cultured DCs). (C) Phagocytic capacity of DCs after cholesterol depletion. (D) Effect of cholesterol depletion on the immune phenotype of DCs. (E) Capacity of DCs to induce T cell proliferation after cholesterol depletion. (F) Capacity of DCs to induce T_reg_ differentiation after cholesterol depletion. For the graph: Mean ± SD, n = 3 to 6 in each group. *P < 0.05; **P < 0.01; ***P < 0.001.

To investigate whether ECM stiffness modulates DC immune function through cholesterol metabolism, this study employed methyl-β-cyclodextrin (M-βCD) to deplete cellular cholesterol. Treatment with 3 mM M-βCD for 4 h significantly reduced cholesterol content without compromising DC viability (Fig. 4A and B).

Subsequent functional assays revealed that cholesterol depletion effectively reversed stiffness-induced aberrant DC maturation. Specifically, M-βCD -treated DCs cultured in stiff hydrogels exhibited markedly attenuated expression of MHC II and the co-stimulatory molecules CD40, CD80, and CD86, alongside a restored phagocytic capacity (Fig. 4C and D). Moreover, cholesterol depletion abrogated the ability of stiffness-primed DCs to stimulate CD4^+^ T cell proliferation (Fig. 4E) and restored their capacity to induce T_reg_ differentiation (Fig. 4F). These findings align with previous reports demonstrating that free cholesterol enhances DC costimulatory molecule expression and CD8^+^ T cell activation [59].

In accordance with a prior study reporting that cholesterol depletion mitigates macrophage inflammatory activation [64], we found that reducing cholesterol reversed stiffness-induced abnormal DC maturation, restored T_reg_ differentiation. While cholesterol depletion broadly disrupts lipid raft assembly and suppresses antigen presentation across myeloid populations in a manner independent of ECM mechanical cues, our study highlights a distinct, mechanosensitive role for cholesterol. Beyond its fundamental structural roles, intracellular cholesterol acts as a critical downstream mechanotransduction effector, translating ECM stiffness cues into altered DC-mediated T cell polarization.

### 3.5 Hepatic fibrosis ECM Stiffness Promoted Cholesterol Accumulation in DCs via AMPK pathway inhibition

**Fig. 5:**
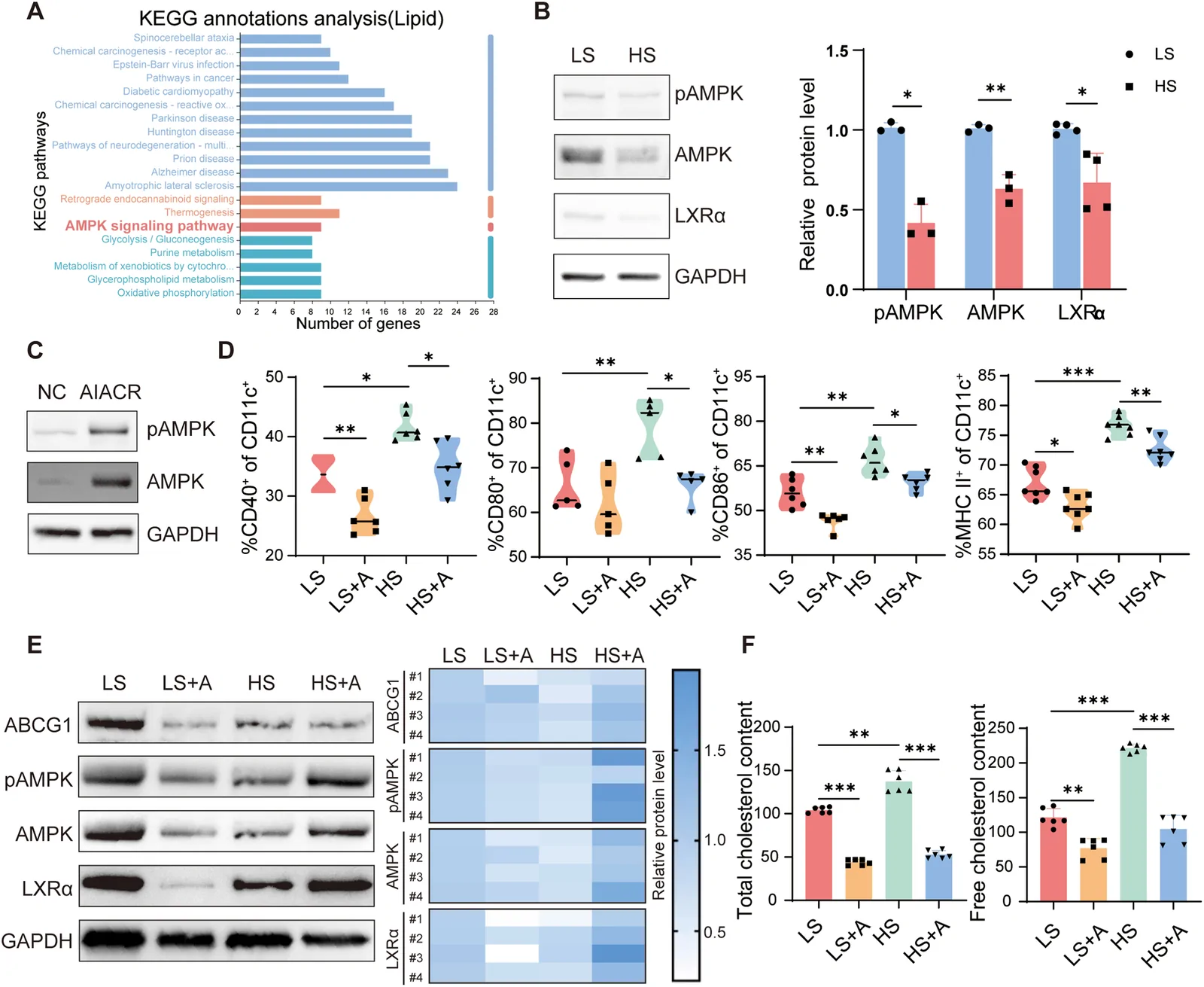
Hepatic Fibrosis ECM Stiffness Promoted Cholesterol Accumulation in DCs by suppressing AMPK Signaling. (A) KEGG annotations analysis of DEGs in lipid metabolism. (B) Expression levels of AMPK-related proteins in DCs treated with different stiffness conditions. (C) Effect of 1 mM AICAR treatment on AMPK expression in DCs. (D) Effect of AICAR treatment on the immune phenotype of DCs. (E) Effect of AICAR treatment on cholesterol efflux-related proteins in DCs. (F) Effect of AICAR treatment on cholesterol content in DCs. For the graph: Mean ± SD, n = 3 to 6 in each group. *P < 0.05; **P < 0.01; ***P < 0.001.

To further elucidate the mechanism underlying stiffness-regulated cholesterol metabolism in DCs, we performed transcriptomic analysis, which revealed that lipid metabolism-related differentially expressed genes were enriched in the AMPK signaling pathway (Fig. 5A). As a master regulator of cellular energy and lipid metabolism, AMPK represents a strong candidate mediator of cholesterol accumulation and aberrant DC activation [65,66]. Previous studies have established that AMPK promotes cholesterol efflux by activating LXRα, which in turn upregulates ABCA1 and ABCG1 to enhance cholesterol transport activity [65,67]. Consistent with this canonical regulatory axis, our data demonstrate that high stiffness represses AMPK activity, concomitant with reduced LXRα and ABCG1 expression and marked intracellular cholesterol retention in DCs (Figs. 5B and 3D–G). To validate the role of AMPK in this mechanotransduction process, we treated DCs with the pharmacological AMPK agonist AICAR (1 mM). CCK-8 and Western blot assays confirmed that this dose robustly elevated AMPK signaling without inducing cytotoxicity (Fig. S4C, Fig. 5C). Crucially, pharmacological AMPK activation effectively reversed the stiffness-induced aberrant DC phenotype. AICAR treatment significantly attenuated upregulation of MHC II and co-stimulatory molecules (CD40, CD80, CD86) in stiffness-primed DCs (Fig. 5D). Concurrently, AMPK activation rescued stiffness-mediated LXRα suppression, recovered ABCG1 expression, and thereby alleviated intracellular cholesterol accumulation (Fig. 5E and F). Collectively, while our pharmacological rescue experiments and protein expression data strongly implicate the AMPK-LXRα-ABCG1 signaling cascade in stiffness-mediated DC dysfunction, we did not perform direct genetic rescue experiments such as LXRα or ABCG1 overexpression. Previous studies identify this axis as a canonical pathway governing cholesterol homeostasis in myeloid cells, consistent with our observation that AMPK activation restores LXRα/ABCG1 expression and mitigates cholesterol accumulation. [[68], [69], [70]]. However, without direct downstream genetic rescue, we cannot exclude the possibility that other AMPK-dependent metabolic branches also contribute to this process. Future studies using conditional knockout models are needed to dissect the exact hierarchical contributions of these downstream effectors.

### 3.6 Systemic inhibition of cholesterol synthesis Ameliorated hepatic fibrosis

**Fig. 6:**
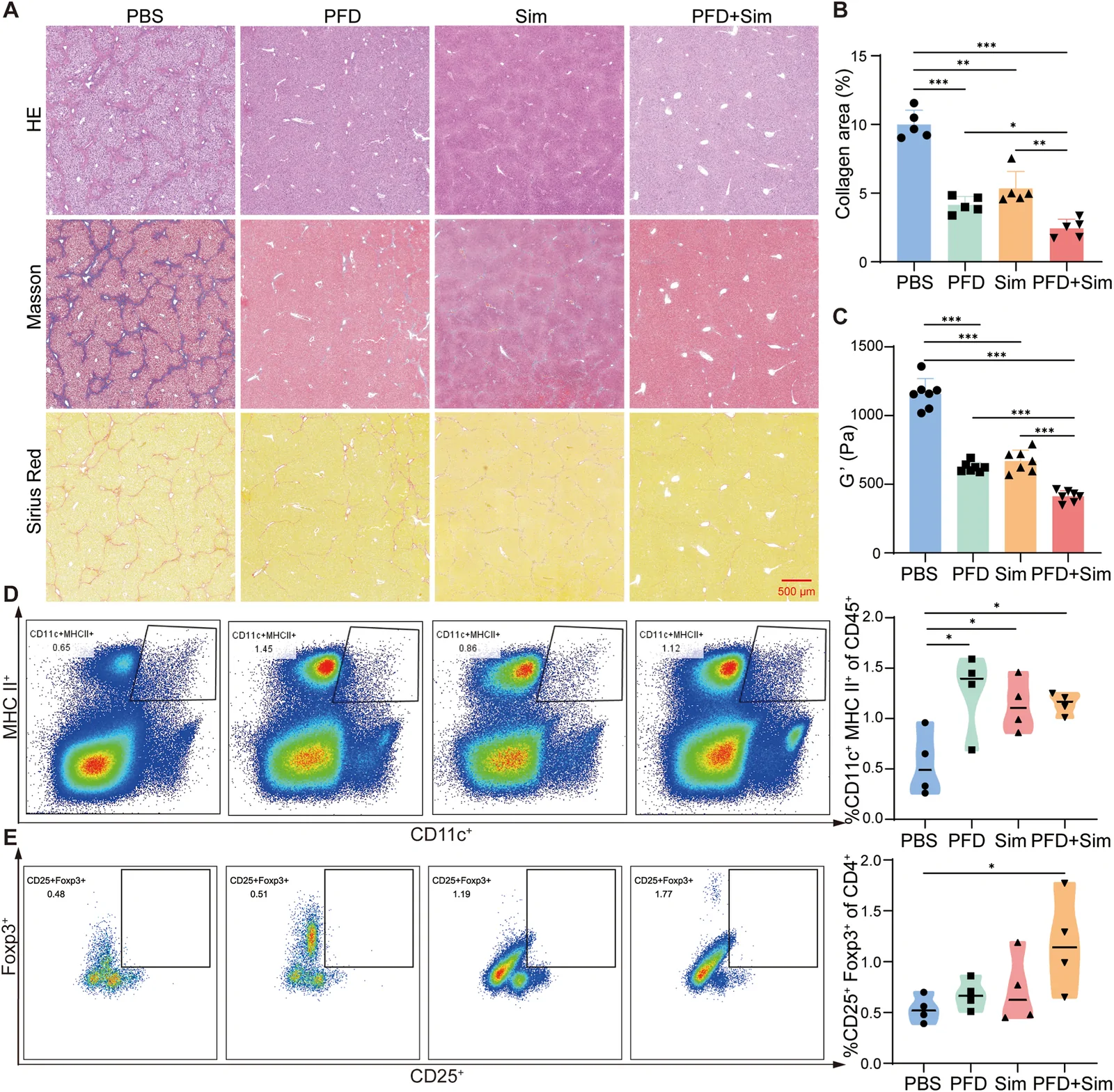
Systemic Inhibition Cholesterol Synthesis Ameliorated Hepatic Fibrosis. (A) Representative images of liver HE, Masson and Sirius Red staining from each treatment group. (B) Quantification of collagen-positive area from Masson staining. (C) Quantification of liver stiffness in mice from each treatment group measured by rheometer. (D) Proportion of DCs in the liver of mice from each treatment group. (E) Proportion of T_reg_ in the liver of mice from each treatment group. Mean ± SD, n = 4 to 7 in each group. *P < 0.05; **P < 0.01; ***P < 0.001. (For interpretation of the references to color in this figure legend, the reader is referred to the Web version of this article.)

To evaluate the therapeutic potential of targeting this mechano-metabolic axis *in vivo*, we treated mice with established hepatic fibrosis using a combination of pirfenidone (PFD) and simvastatin (Sim), a selective HMGCR inhibitor that specifically suppresses cholesterol synthesis. The results showed that combination therapy yielded superior efficacy in reducing hepatic collagen deposition and liver stiffness (Fig. 6A–C), consistent with their established mechanisms of action. Pirfenidone inhibits TGF-β/Smad signaling to limit collagen synthesis [71,72], while simvastatin inhibits HMGCR to lower cholesterol and block HSC activation [73,74]. Subsequently, flow cytometric analysis revealed that PFD, Sim, and combination treatment (PFD + Sim) all increased the proportion of hepatic DCs relative to PBS-treated controls (Fig. 6D). Notably, only the PFD + Sim combination significantly enhanced IL-10 production by DCs and expanded the proportion of T_reg_ in the liver (Fig. 6E, Fig. S5F). This synergistic immunomodulatory effect is associated with Sim-mediated cholesterol reduction, which is consistent with the restoration of the tolerogenic function of DCs to promote IL-10 secretion [75,76], acting in concert with PFD-induced matrix softening. This creates a favorable microenvironment for T_reg_ differentiation, ultimately enhancing the anti-fibrotic efficacy of PFD.

**Fig. 7:**
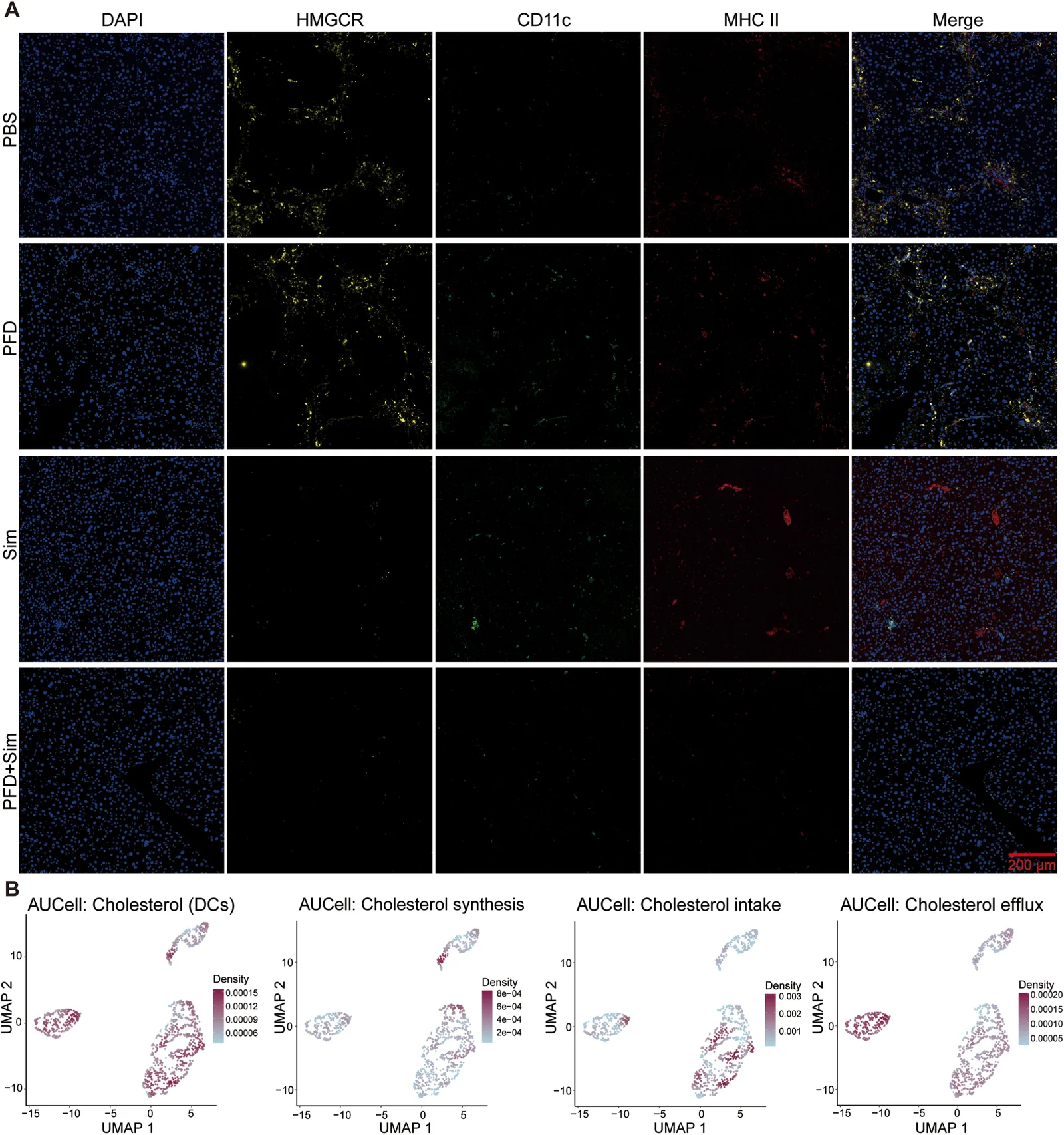
Simvastatin Regulates Hepatic DC Cholesterol Metabolism. (A). Representative immunofluorescence staining of HMGCR expression in hepatic DCs from mice. (B). Cholesterol metabolism scores of DCs in single-cell RNA sequencing of liver tissue.

To verify the regulatory effects of Sim on hepatic DCs, we performed immunofluorescence staining of liver tissues. Sim robustly suppressed HMGCR expression in DCs, while DCs from mice receiving PFD alone maintained high levels of HMGCR (Fig. 7A). Notably, simvastatin was administered systemically in this study. Thus, we cannot fully distinguish direct DC-intrinsic effects from indirect microenvironmental alterations mediated by other hepatic cell populations. Even so, the observed reduction of DC HMGCR upon systemic treatment corroborates our *in vitro* mechanometabolic results. We have quantified HMGCR fluorescence intensity and DC-HMGCR colocalization across biological replicates (Fig. S5B and S5C). Collectively, these results indirectly support the notion that systemic simvastatin treatment lowers cholesterol content in hepatic DCs. To further explore the clinical relevance of these findings, we constructed a cholesterol metabolism disorder score in the DC subsets using human single-cell transcriptomic data. Notably, conventional DC1 (cDC1) displayed elevated cholesterol uptake scores, cDC2 exhibited the highest cholesterol synthesis scores, and plasmacytoid DC (pDC) showed significantly increased cholesterol efflux scores (Fig. 7B). These findings indicate that targeting cholesterol metabolism in DCs could provide a precise therapeutic strategy for treating hepatic fibrosis.

While we define a core mechano-metabolic axis whereby pathological ECM stiffness drives DC immune dysfunction, the upstream mechanosensing machinery that initiates this cascade remains uncharacterized. Further experiments are required to verify whether canonical mechanotransducers,such as Piezo1, integrin adhesion complexes and YAP/TAZ, act as upstream regulators of the AMPK-LXRα-ABCG1 pathway. Another limitation lies in the heterogeneity of hepatic DC subsets. Single-cell transcriptomic data reveal distinct cholesterol metabolic signatures among hepatic cDC1, cDC2 and pDC subsets under fibrotic conditions. However, we have not validated these subset-specific alterations *in vitro* or *in vivo*. Because we focused on the overarching mechano-metabolic axis within bulk DC populations, it remains unclear whether all DC subsets contribute equally to fibrosis progression or whether one specific subset acts as the dominant driver. Future studies combining subset-specific fluorescence-activated cell sorting (FACS) with downstream functional assays (e.g., subset-specific cholesterol quantification, cytokine profiling, and T cell priming) will be essential to define their distinct roles in stiffness-triggered immune dysfunction. Finally, the cell-type specificity and clinical translational potential of cholesterol-targeted therapeutic strategies remain to be validated in sophisticated preclinical fibrotic models and well-powered human patient cohorts.

## 4 Conclusions

Our research identified a mechano-metabolic axis in hepatic fibrosis. ECM stiffness in fibrotic liver reprograms DC cholesterol metabolism via the AMPK-LXRα-ABCG1 signaling axis, driving abnormal DC maturation and fibrogenesis. Targeting cholesterol accumulation in DCs may enhance antifibrotic pharmacotherapy. Thus, modulating DC cholesterol metabolism holds promise as an effective strategy to restore immune homeostasis and optimize liver fibrosis treatment. This mechanometabolic target holds potential for developing biomaterial-based or drug-based anti-fibrotic therapies.

## CRediT authorship contribution statement

**Xianlin Zeng:** Writing – original draft, Visualization, Validation, Methodology, Investigation, Formal analysis, Data curation, Conceptualization. **Pan Luo:** Validation, Methodology, Investigation, Formal analysis, Data curation. **Cuifang Wu:** Software, Methodology, Formal analysis, Data curation. **Jinhua Long:** Supervision, Project administration, Funding acquisition. **Shuai Zhang:** Conceptualization. **Yun Wang:** Supervision. **Lijing Teng:** Methodology. **Honghong Zhang:** Investigation. **Deqiao Fan:** Data curation. **Zuquan Hu:** Supervision, Resources, Project administration. **Pu Xu:** Writing – review & editing, Writing – original draft, Validation, Investigation, Funding acquisition. **Zhu Zeng:** Writing – review & editing, Supervision, Resources, Project administration, Funding acquisition, Conceptualization.

## Declaration of competing interest

The authors declare that they have no known competing financial interests or personal relationships that could have appeared to influence the work reported in this paper.

## Data Availability

Data will be made available on request.

## References

[1] Ginès P., Krag A., Abraldes J.G. (2021 Oct 9). Liver cirrhosis. Lancet (London, England).

[2] Carvalho A.M., Bansal R., Barrias C.C. (2024 Jan). The material world of 3D-Bioprinted and microfluidic-chip models of human liver fibrosis. Adv. Mater..

[3] European Association for the Study of the Liver (2025 Aug). EASL clinical practice Guidelines on the management of hepatitis B virus infection. J. Hepatol..

[4] Peiseler M., Schwabe R., Hampe J. (2022 Oct). Immune mechanisms linking metabolic injury to inflammation and fibrosis in fatty liver disease - novel insights into cellular communication circuits. J. Hepatol..

[5] Zhang N., Yao H., Zhang Z. (2023 Feb). Ongoing involvers and promising therapeutic targets of hepatic fibrosis: the hepatic immune microenvironment. Front. Immunol..

[6] Huby T., Gautier E.L. (2022 Jul). Immune cell-mediated features of non-alcoholic steatohepatitis. Nat. Rev. Immunol..

[7] Tacke F., Puengel T., Loomba R. (2023 Aug). An integrated view of anti-inflammatory and antifibrotic targets for the treatment of NASH. J. Hepatol..

[8] Tao X., Zhang R., Du R. (2022 May). EP3 enhances adhesion and cytotoxicity of NK cells toward hepatic stellate cells in a murine liver fibrosis model. J experimental Med.

[9] Wang Y., Wang J., Zhang J. (2024 Jun 7). Stiffness sensing via Piezo1 enhances macrophage efferocytosis and promotes the resolution of liver fibrosis. Sci. Adv..

[10] Xu Y., Liu F., He D. (2023 Mar). Monocyte-derived immature dendritic cells negatively regulate hepatic stellate cells in vitro by secreting IL-10. Immunobiology.

[11] Hu W., Wang Y., Chen J. (2022 Mar). Regulation of biomaterial implantation-induced fibrin deposition to immunological functions of dendritic cells. Mater. Today Bio.

[12] Jiao J., Sastre D., Fiel M.I. (2012 Jan). Dendritic cell regulation of carbon tetrachloride-induced murine liver fibrosis regression. Hepatology.

[13] Friedline R.H., Noh H.L., Suk S. (2024 Jun 29). IFNγ-IL12 axis regulates intercellular crosstalk in metabolic dysfunction-associated steatotic liver disease. Nat. Commun..

[14] Lukacs-Kornek V., Schuppan D. (2013 Nov). Dendritic cells in liver injury and fibrosis: shortcomings and promises. J. Hepatol..

[15] Xu Y.D., Cheng M., Shang P.P. (2022 Mar). Role of IL-6 in dendritic cell functions. J. Leukoc. Biol..

[16] English K., Kwan R., Holz L.E. (2024 Feb 10). A hepatic network of dendritic cells mediates CD4 T cell help outside lymphoid organs. Nat. Commun..

[17] Wang B., Yang L., Yuan X. (2024 Jul). Roles and therapeutic targeting of dendritic cells in liver fibrosis. J. Drug Target..

[18] Xu Z., Zhang S., Zhang X. (2025 Oct 15). Cyclized polyacrylonitrile nanoparticles: enhanced intratumoral delivery by photocatalysis and collagen denaturation. J. Colloid Interface Sci..

[19] Forsgren M.F., Nasr P., Karlsson M. (2020 Jul). Biomarkers of liver fibrosis: prospective comparison of multimodal magnetic resonance, serum algorithms and transient elastography. Scand. J. Gastroenterol..

[20] Mennens S.F.B., Bolomini-Vittori M., Weiden J. (2017 Dec 13). Substrate stiffness influences phenotype and function of human antigen-presenting dendritic cells. Sci. Rep..

[21] Lee M., Du H., Winer D.A. (2022 Nov). Mechanosensing in macrophages and dendritic cells in steady-state and disease. Front. Cell Dev. Biol..

[22] Sapudom J., Alatoom A., Mohamed W.K.E. (2020 Sep 15). Dendritic cell immune potency on 2D and in 3D collagen matrices. Biomater. Sci..

[23] Luo J., Yang H., Song B.L. (2020 Apr). Mechanisms and regulation of cholesterol homeostasis. Nat. Rev. Mol. Cell Biol..

[24] Xiao M., Xu J., Wang W. (2023 Sep). Functional significance of cholesterol metabolism in cancer: from threat to treatment. Exp. Mol. Med..

[25] Zuo J., Yan H., Qin S. (2024 Oct). Extracellular vesicles in cancer drug resistance: mechanistic insights and therapeutic implications. MEDCOMM - Oncology.

[26] Jacobs C.F., Peters F.S., Camerini E. (2025 May). Cholesterol homeostasis and lipid raft dynamics at the basis of tumor-induced immune dysfunction in chronic lymphocytic leukemia. Cell. Mol. Immunol..

[27] Westerterp M., Gautier E.L., Ganda A. (2017 Jun 6). Cholesterol accumulation in dendritic cells links the inflammasome to acquired immunity. Cell Metab..

[28] Zhang X., Chen Y., Sun G. (2024 Nov). Farnesyl pyrophosphate potentiates dendritic cell migration in autoimmunity through mitochondrial remodelling. Nat. Metab..

[29] Li N., Zhang X., Zhou J. (2022 Sep). Multiscale biomechanics and mechanotransduction from liver fibrosis to cancer. Adv. Drug Deliv. Rev..

[30] Long Y., Niu Y., Liang K. (2022 Jan). Mechanical communication in fibrosis progression. Trends Cell Biol..

[31] Ahodantin J., Wu J., Funaki M. (2024 May). Siglec-H(-/-) plasmacytoid dendritic cells protect against acute liver injury by suppressing IFN-γ/Th1 response and promoting IL-21(+) CD4 T cells. Cellular and molecular gastroenterology and hepatology.

[32] Huang X., He T., Liang X. (2024 Mar). Advances and applications of nanoparticles in cancer therapy. MEDCOMM-Oncology.

[33] Taru V., Szabo G., Mehal W. (2024 Nov). Inflammasomes in chronic liver disease: hepatic injury, fibrosis progression and systemic inflammation. J. Hepatol..

[34] Yun H.J., Suh Y.J., Kim Y.B. (2022 Nov 13). Hepatocyte DAX1 deletion exacerbates inflammatory liver injury by inducing the recruitment of CD4(+) and CD8(+) T cells through NF-κB p65 signaling pathway in mice. Int. J. Mol. Sci..

[35] Fu J., Chen T., Fang X. (2025 Oct). Immune cell subsets as therapeutic targets in liver fibrosis: from mechanisms to translational applications. Biochemical and biophysical research communications.

[36] Aki D., Hayakawa T., Srirat T. (2024 Oct 15). The Nr4a family regulates intrahepatic Treg proliferation and liver fibrosis in MASLD models. J. Clin. Investig..

[37] Zhang B., Wang J., Liu M. (2022 Oct). IL-10 regulates Th17 response to inhibit hepatobiliary injury caused by Clonorchis sinensis infection in C57BL/6J mice. Front. Cell. Infect. Microbiol..

[38] Wang R., Liang Q., Zhang Q. (2024 Dec). Ccl2-Induced regulatory T cells balance inflammation through macrophage polarization during liver reconstitution. Adv. Sci..

[39] Andrews T.S., Nakib D., Perciani C.T. (2024 May). Single-cell, single-nucleus, and spatial transcriptomics characterization of the immunological landscape in the healthy and PSC human liver. J. Hepatol..

[40] Zhang X., Lou J., Bai L. (2017 Feb 25). Immune regulation of intrahepatic regulatory T cells in fibrotic livers of mice. Med. Sci. Monit. : Int. Med. J. Exp. Clin. Res..

[41] Yu Y.R., O'Koren E.G., Hotten D.F. (2016 Mar). A protocol for the comprehensive flow cytometric analysis of immune cells in normal and inflamed Murine non-lymphoid tissues. PLoS One.

[42] Lambooij J.M., Tak T., Zaldumbide A. (2024 Apr). OMIP‐104: a 30‐color spectral flow cytometry panel for comprehensive analysis of immune cell composition and macrophage subsets in mouse metabolic organs. Cytometry A.

[43] Tuominen I., Fuqua B.K., Pan C. (2021 Aug). The genetic Architecture of carbon tetrachloride-induced liver fibrosis in mice. Cellular and molecular gastroenterology and hepatology.

[44] Fujii T., Fuchs B.C., Yamada S. (2010 Jul). Mouse model of carbon tetrachloride induced liver fibrosis: histopathological changes and expression of CD133 and epidermal growth factor. BMC Gastroenterol..

[45] Mueller S., Sandrin L. (2010 May 25). Liver stiffness: a novel parameter for the diagnosis of liver disease. Hepatic Med..

[46] Desai S.S., Tung J.C., Zhou V.X. (2016 Jul). Physiological ranges of matrix rigidity modulate primary mouse hepatocyte function in part through hepatocyte nuclear factor 4 alpha. Hepatology.

[47] Kostallari E., Wei B., Sicard D. (2022 Feb 1). Stiffness is associated with hepatic stellate cell heterogeneity during liver fibrosis. Am. J. Physiol. Gastrointest. Liver Physiol..

[48] Kostallari E., Wei B., Sicard D. (2021 Dec). Stiffness is associated with hepatic stellate cell heterogeneity during liver fibrosis. Am. J. Physiol. Gastrointest. Liver Physiol..

[49] Kawamura N., Imajo K., Kalutkiewicz K.J. (2022 Dec). Influence of liver stiffness heterogeneity on staging fibrosis in patients with nonalcoholic fatty liver disease. Hepatology.

[50] Saluja S.S., Hanlon D.J., Sharp F.A. (2014 Nov). Targeting human dendritic cells via DEC-205 using PLGA nanoparticles leads to enhanced cross-presentation of a melanoma-associated antigen. International journal of nanomedicine.

[51] Cho K.J., Ishido S., Eisenlohr L.C. (2020 Mar 15). Activation of dendritic cells alters the mechanism of MHC class II antigen presentation to CD4 T cells. J. Immunol..

[52] Heinl P.V., Graulich E., Weigmann B. (2024 Oct). IL-10-modulated dendritic cells from birch pollen- and hazelnut-allergic patients facilitate Treg-mediated allergen-specific and cross-reactive tolerance. Allergy.

[53] Jamison B.L., Lawrance M., Wang C.J. (2024 Nov 26). An IL-2 mutein increases regulatory T cell suppression of dendritic cells via IL-10 and CTLA-4 to promote T cell anergy. Cell Rep..

[54] Choi Y., Kwon J.E., Cho Y.K. (2021 Nov 30). Dendritic cell migration is tuned by mechanical stiffness of the confining space. Cells.

[55] Chakraborty M., Chu K., Shrestha A. (2021 Jan 12). Mechanical stiffness controls dendritic cell metabolism and function. Cell Rep..

[56] Saleem M., Morlot S., Hohendahl A. (2015 Feb 19). A balance between membrane elasticity and polymerization energy sets the shape of spherical clathrin coats. Nat. Commun..

[57] Pontes B., Monzo P., Gole L. (2017 Sep 4). Membrane tension controls adhesion positioning at the leading edge of cells. J. Cell Biol..

[58] Lühr J.J., Alex N., Amon L. (2020 Nov). Maturation of monocyte-derived DCs leads to increased cellular stiffness, higher membrane fluidity, and changed lipid composition. Front. Immunol..

[59] Zhao H., Yu Y., Wang Y. (2022 Dec). Cholesterol accumulation on dendritic cells reverses chronic hepatitis B virus infection-induced dysfunction. Cell. Mol. Immunol..

[60] Tall A.R., Yvan-Charvet L. (2015 Feb). Cholesterol, inflammation and innate immunity. Nat. Rev. Immunol..

[61] Xu Y., Chen Y., Zhang J. (2025 Mar). RNASET2 deficiency induces hepatocellular carcinoma metastasis through cholesterol-triggered MET activation. Adv. Sci..

[62] Chen J., Wu Z., Zhang Z. (2024 Nov). Apparent diffusion coefficient and tissue stiffness are associated with different tumor microenvironment features of hepatocellular carcinoma. Eur. Radiol..

[63] Jiang W., Jin W.L., Xu A.M. (2024 Mar). Cholesterol metabolism in tumor microenvironment: cancer hallmarks and therapeutic opportunities. Int. J. Biol. Sci..

[64] Zimmer S., Grebe A., Bakke S.S. (2016 Apr 6). Cyclodextrin promotes atherosclerosis regression via macrophage reprogramming. Sci. Transl. Med..

[65] Lee M.K.S., Cooney O.D., Lin X. (2022 Jul). Defective AMPK regulation of cholesterol metabolism accelerates atherosclerosis by promoting HSPC mobilization and myelopoiesis. Mol. Metabol..

[66] Hu H.J., Wang X.H., Zhang T.Q. (2022 Dec). PLK1 promotes cholesterol efflux and alleviates atherosclerosis by up-regulating ABCA1 and ABCG1 expression via the AMPK/PPARγ/LXRα pathway. Biochim. Biophys. Acta Mol. Cell Biol. Lipids.

[67] He X.W., Yu D., Li W.L. (2016 Oct). Anti-atherosclerotic potential of baicalin mediated by promoting cholesterol efflux from macrophages via the PPARγ-LXRα-ABCA1/ABCG1 pathway. Biomed. Pharmacother..

[68] Li X., Zhang X., Wei N. (2025 Oct). Total alkaloids from Coptis chinensis Franch ameliorate hyperlipidemia and hepatic steatosis via dual pathway modulation of AMPK/SREBP-1c and PPARα/LXRα in mice. Front. Pharmacol..

[69] Clark A.T., Russo-Savage L., Ashton L.A. (2025 Jan 28). A mutation in LXRα uncovers a role for cholesterol sensing in limiting metabolic dysfunction-associated steatohepatitis. Nat. Commun..

[70] Wang Y., Zhang Y., Che X. (2026 Sep 15). Lycium barbarum glycopeptide alleviates diabetic retinopathy and hepatic lipid disorder via modulation of the LXR-α/ABC transporter axis. J. Ethnopharmacol..

[71] Wang W., Gao Y., Chen Y. (2025 Jun). TGF-β inhibitors: the future for prevention and treatment of liver fibrosis?. Front. Immunol..

[72] Komiya C., Tanaka M., Tsuchiya K. (2017 Mar 17). Antifibrotic effect of pirfenidone in a mouse model of human nonalcoholic steatohepatitis. Sci. Rep..

[73] Marrone G., Russo L., Rosado E. (2013 Jan). The transcription factor KLF2 mediates hepatic endothelial protection and paracrine endothelial-stellate cell deactivation induced by statins. J. Hepatol..

[74] Trebicka J., Hennenberg M., Laleman W. (2007 Jul). Atorvastatin lowers portal pressure in cirrhotic rats by inhibition of RhoA/Rho-kinase and activation of endothelial nitric oxide synthase. Hepatology.

[75] Belabed M., Park M.D., Blouin C.M. (2025 Feb). Cholesterol mobilization regulates dendritic cell maturation and the immunogenic response to cancer. Nat. Immunol..

[76] Hu M.X., Li J.Q., Zhang H.W. (2025 Mar). 1-benzyl-6-nitro-4-phenyl-4-(methoxycarbonyl)-2(1H)-pyridinone, a novel pirfenidone derivative, alleviate hepatic fibrosis through T cells. Biomed. Pharmacother..

