# Supplementary material for: Hepatic fibrosis extracellular matrix stiffness induces abnormal maturation of dendritic cell via AMPK inhibition-mediated cholesterol accumulation
Source: Mater Today Bio. 2026 Sep 1;40:103606. doi: 10.1016/j.mtbio.2026.103606 (PMC13586880; doi:10.1016/j.mtbio.2026.103606)
Supplement: Supplementary file 1 [file mmc1.docx]

Supplementary Materials for

**Hepatic Fibrosis Extracellular Matrix Stiffness Induces Abnormal Maturation of Dendritic Cells via AMPK-promoted Cholesterol Accumulation**

Xianlin Zeng et al.

*Corresponding author: Zhu Zeng, [zengzhu@gmc.edu.cn](mailto:zengzhu@gmc.edu.cn); Pu Xu, [xupu202208@163.com](mailto:xupu202208@163.com); Zuquan Hu, [huzuquan@gmc.edu.cn](mailto:huzuquan@gmc.edu.cn).

**This PDF file includes:**

Figs. S1 to S5

Tables S1

**Supplement Figures**

**Fig. S1. Characteristics of** **Hepatic Fibrotic Tissue.**

(A) UMAP visualization of immune cells from mouse liver tissue analyzed by single‑cell RNA sequencing. (B) Annotation of immune cells subsets in liver tissues. (C) Immune cell clustering in liver tissue using signature genes. Myeloid cells were extracted from total immune cells, followed by identification of dendritic cells within the myeloid subset. (D) Representative images of liver. (E) Quantification of collagen-positive area from Masson staining. (F) Concentrations of ALT, and AST in liver tissues. (G) Stiffness analysis of mouse liver tissue using atomic force microscopy (AFM). (H) Stiffness analysis of mouse liver tissue using rheometer.

**Fig. S2. Flow Cytometric Analysis of Immune Cells in Liver Tissue.**

(A) Flow cytometric gating strategy for murine hepatic DCs and T_reg_. (B) Proportion of CD4^+^ or CD8^+^ cells in liver tissues. (C) Concentrations of IL-6 in liver tissues. (D) Flow cytometric gating strategy for in *vitro* DCs marker. (E) Flow cytometric gating strategy for in *vitro* T_reg_.

**Fig. S3. The Influence of Hepatic ECM Stiffness on DC-Mediated T Cells Differentiation.**

(A) Representative flow cytometry plots of Th_1_, Th_2_, and Th_17_ differentiation by DCs treated with different stiffness. (B) Effect of DCs treated with different stiffness on Th_1_, Th_2_ and Th_17_ differentiation. (C) Effect of different stiffness on osmotic fragility of DCs (Y-axis: The OD value reflected cell viability). (D) Effect of different stiffness on membrane fluidity of DCs. (E) Phagocytic capacity of DCs treated with different stiffness. (F) Cytokine secretion profiles in supernatants of T cells cultured with DCs exposed to different stiffness.

**Fig. S4. The Effect of Cholesterol Depletion on Stiffness-Treated DCs.**

(A) Effect of different stiffness on lipid raft content in DCs. (B) Capacity of DCs to induce Th_1_, Th_2_ and Th_17_ differentiation after cholesterol depletion. (C) Effect of AICAR treatment at different concentrations on DCs viability.

**Fig. S5. Inhibition of Cholesterol Synthesis Ameliorated Hepatic Fibrosis**

(A) Quantification of total cholesterol and free cholesterol levels in mouse liver homogenates (μmol/μg). (B) Quantification of HMGCR fluorescence intensity in hepatic DCs after drug treatment. (C) Colocalization analysis of CD11c and HMGCR proteins (Red lines indicate the selected regions for quantitative colocalization calculation). (D) Stiffness analysis of mouse liver tissue using rheometer. (E, F) Expression levels of CD80 and CD86, and IL-10 in hepatic DCs. (G) Cholesterol metabolism scores of immune cells in single-cell RNA sequencing of liver tissue.

**Table S1. Primers used in qRT-PCR**

| Gene | Forward Primer | Reverse Primer |
| --- | --- | --- |
| GAPDH | ACGGCAAATTCAACGGCACAG | ACACCAGTAGACTCCACGACATAC |
| HMGCR | GCAGATGCTAGGTGTTCAAGGAG | CAGCCATCACAGTGCCACATAC |
| APOE | GGCGGCTGGAGGAAGTGG | GGCGTATTTGCTGGGTCTGTTC |
| NPC1L1 | GGCTGGCTCTCATCATCATCTTC | GCTGTCTTGTTCTTGTTCCTGTTG |
| LDLR | TGAGGTTCCTGTCCATCTTCTTCC | GATGTTCTTCAGCCGCCAGTTC |
| ABCG1 | TGCTGCTGCCTCACCTCAC | TCTCGTCTGCCTTCATCCTTCTC |
| ABCA1 | GAAGGAGGCTCGGCTGAAGG | GAGGGATGAGGCTGCTAACAAAC |
| ACAT1 | TCTCACGGCAGGAACAGGATAC | TCATCTTCTTTCACCACCACATCTG |
| ACAT2 | GCTGCTGCTGTGGTCCTTATG | GCTTGCTTTATGGCTGGAATTGG |
